# Satisfaction with urban trees associates with tree canopy cover and tree visibility around the home

**DOI:** 10.1038/s42949-023-00119-8

**Published:** 2023-06-23

**Authors:** Camilo Ordóñez, S. M. Labib, Lincoln Chung, Tenley M. Conway

**Affiliations:** 1 Department of Geography, Geomatics and Environment, University of Toronto at Mississauga, 3359 Mississauga Road, Mississauga, ON L5L 1C6 Canada; 2 Department of Human Geography and Spatial Planning, Faculty of Geosciences, Utrecht University, 3584 CB Utrecht, The Netherlands

**Keywords:** Urban ecology, Psychology and behaviour

## Abstract

Many world cities want to expand the number of urban trees. How this expansion occurs should consider what people expect from trees based on how they experience and perceive these trees. Therefore, we need a better understanding of how people perceptually respond to urban tree abundance. This research examined whether people’s satisfaction with urban trees and satisfaction with the management of those trees were related to objective measures of greenery such as the Normalized Difference Vegetation Index (NDVI), percent tree canopy cover, and the Viewshed Greenness Visibility Index (VGVI) for trees. We used a demographically and geographically representative survey of 223 residents in Toronto, Canada, and calculated NDVI, canopy cover, and VGVI at three neighbourhood sizes. We analysed the data using generalized linear regression. We found that canopy cover and VGVI had a positive association with satisfaction with urban trees. The associations were comparatively stronger at larger neighbourhood scales than at smaller scales. There were no statistically significant associations with NDVI or satisfaction with the management of urban trees.

## Introduction

Urban trees support urban sustainability by contributing to the environmental, economic, and social health of urban communities^1,2^ through provision of key ecosystem services^3,4^. Thus, enhancing and protecting urban trees is necessary to make cities inclusive, safe, and resilient (https://sdgs.un.org/goals/goal11). This has been recognized through UN-led initiatives (https://habitat3.org) down to numerous local initiatives to plant more trees in urban areas^3^. The success of such initiatives depends not only on technical knowledge regarding maintenance, protection, and planting, but also developing and implementing policies that address the needs and desires of the public, considering the diverse perspectives and experiences people have with urban trees.

Despite efforts to grow urban tree populations, these populations are decreasing globally^5^ due to pressures from urban (re-)development^6^ and climate change, which may exacerbate existing environmental stressors, such as heat stress, drought, and pest and diseases^7^. Moreover, the current distribution of urban trees is often uneven, resulting in inequalities in experiences with them and the services they provide. For example, historic raced-based housing discrimination in the US is related to urban trees inequities across different neighbourhoods today^8^ which can result in lower levels of wellbeing and other health indicators due to reduced exposure to urban trees^9^.

While greater exposure to urban trees might be beneficial for health and wellbeing, increasing trees without accounting for how the community experiences and perceives them may result in disconnected, and possibly detrimental, outcomes. Urban trees provide critical regulating and provisioning ecosystem services, such as air pollution regulation, noise mitigation, and heat mitigation^4,10^, regardless of what people may feel or think about them, but they may fail to provide other services that are also desired by the community, such as aesthetic and cultural value, or to mitigate disservices, such as allergies or windthrow^11^. Indeed, urban trees that do not meet people’s expectations, may result in lower community support for tree planting initiatives^12^.

To support successful enhancement efforts and address existing inequities, understanding how specific community perception responses relate to specific ecological structures around a person’s living environment is needed. Perception refers to how people mentally process the information from the environment around them. Perception can be influenced by several biological (e.g., how we sense), physical (i.e., what we sense, including specific objects or visual fields), and socio-cultural factors (i.e., how we interpret inputs of what we sense). How to account for these factors in the research on people’s perceptions of urban nature depends on the specific perception responses (i.e., specific cognitive constructs, such as values, beliefs, attitudes, and preferences; see *Methods* section) and the specific ecological structures. Many studies have been conducted on people’s universal preferences of the urban environment and the role of nature visuals in these preferences, with the aim of integrating nature into this environment in broad terms^13,14^. However, fewer studies have been conducted on people’s perception responses to specific ecological structures (e.g., values, beliefs, attitudes, and preferences associated with the abundance, diversity, arrangement, visibility, or condition of urban trees)^15^.

A key perception response is satisfaction, which is the discrepancy between expectation and experience, and a key ecological structure of the urban environment is urban trees. Satisfaction with urban trees is a useful perceptual response, as it helps understand whether people’s experiences of existing trees and management initiatives align with their expectations^16,17^. While the premise that more trees may lead to greater satisfaction is intuitive, this has not been explicitly examined at multiple spatial scales that people experience these urban trees. Previous work has shown only a weak association between presence of trees and satisfaction with them, based on the correlation between average satisfaction and mean urban tree canopy cover at the city scale^18^. However, it remains unclear whether the abundance of trees at relatively finer spatial scales (e.g., neighbourhood scale) result in varying levels of community satisfaction with trees.

Although perception response measures specific to urban trees may be valued by practitioners, they are usually not considered in urban tree management^19^. Traditional measures are focused on technical aspects or biophysical conditions, such as the maintenance, health, diversity, arrangement, and distribution of urban trees^20^, or the consequences of these conditions, such as ecosystem services and disservices^21^. Social measures of economic (e.g., property values^22^) and sociological conditions (e.g., crime rates^23^) have been extensively examined in relation to urban tree characteristics^8^, but these measures typically do not capture community perception responses to urban trees, including satisfaction. Few studies have paired specific community perception responses with the characteristics of urban trees (i.e., abundance, diversity, arrangement, condition)^15,18^. Recent research has suggested that people’s preferences for biodiverse landscapes can be influenced by how biodiverse these landscapes are^24,25^, and people’s preferences for places with or without trees can be influenced by the absence or presence of trees^26^. There are also plenty of studies that assess how people perceive the benefits/services or costs/disservices provided by urban trees in general terms^27–29^. However, there is no evidence that more abundant and visible urban trees may lead to greater satisfaction with urban trees.

A challenge is that there are various methodologies for measuring urban tree abundance and visibility, and different spatial scales of analysis. These different measures and scales may not align with how people perceive trees. For example, many studies have measured tree abundance using satellite image-derived Normalized Difference Vegetation Index (NDVI), which reflects the abundance of trees and all other vegetation, while others consider tree canopy cover. These approaches capture “bird’s eye” measures^9,30^. Such measures correlate positively with city-wide wellbeing and physical health indicators^1,2,31^, but it is unclear if these top-down measures are related to satisfaction with trees.

Recently emerging urban greenness measures that may more accurately represent the experiences people have with urban trees based on what is visible at eye-level^32,33^. These “eye-level” greenness measures include the Green View Index, which uses street view data^34^ and the Viewshed Greenness Visibility Index (VGVI), which uses digital elevation data^33^. While these measures also indicate positive associations with mental health conditions^34^ and subjective wellbeing^35^, the associations between eye-level measures of tree visibility and satisfaction with trees have not yet been investigated.

This research examined whether people’s satisfaction with trees and satisfaction with their management were related to different types of neighbourhood-level greenness measures. We addressed three research questions: (1) is there an association between residents’ level of satisfaction with urban trees and greenness measures? (2) is there an association between residents’ level of satisfaction with the management of urban trees and greenness measures? and (3) does the magnitude or strength of the associations change as the neighbourhood size changes?

By assessing both satisfaction with urban trees and satisfaction with the management of these trees (questions 1 and 2) we aimed to account for different aspects of community satisfaction. On the one hand, people can be satisfied with the characteristics of the ecological structures around their living environment, such as their abundance, diversity, and distribution. On the other hand, people can be satisfied with how people make decisions about these ecological structures, such as investment, responsiveness, and maintenance, with such decisions directly impacting the abundance, diversity, and distribution of urban trees. By asking about both these aspects we can complementarily assess the different dimensions of community satisfaction with urban trees.

To answer these questions, we collected data on people’s perception responses through an online panel survey in the City of Toronto, Canada. The survey included questions about people’s level of satisfaction with urban trees and people’s level of satisfaction with urban tree management^18^. To account for cognitive, social-ecological context, and demographic influences on these perceptions, we also collected data on people’s level of nature relatedness^36^, level of tree knowledge^37^, and various social-ecological context and demographic variables, including age, education, year living in the neighbourhood, and cultural identity (see Methods).

Using the postal codes of respondents from the survey, we calculated three neighbourhood-level greenness measures. We focused on NDVI, percent tree canopy cover, and VGVI for trees only. Each measure was calculated based on three buffer sizes around the postal code: 100 m, 300 m, and 500 m. We then analysed these data using regression-based approaches. In our analysis, we chose to control for these cognitive, social-ecological context, and demographic factors so we could focus on the relationship between people’s subjective satisfaction and objective neighbourhood-level greenness measures (see Methods).

We hypothesized that there would be a comparatively stronger positive relationship (i.e., higher correlation and coefficient values) between the residents’ level of satisfaction and VGVI than with NDVI and canopy cover, because VGVI is more reflective of people’s eye-level visibility of urban trees than the other two top-down measures. We also hypothesized that there would be a comparatively stronger positive relationship between these measures at the larger neighbourhood scales, as the greenness measures of trees cover a larger spatial extent and include areas with greater tree numbers, thus at larger spatial scales of analysis, the statistics may indicate comparatively stronger correlations due to the spatial aggregation effect^30,38^ These hypotheses also have a psycho-social basis, considering the standard walkable distance used in urban greenness and physical health studies^31^.

## Results

### Overview of survey responses

**Fig. 1:**
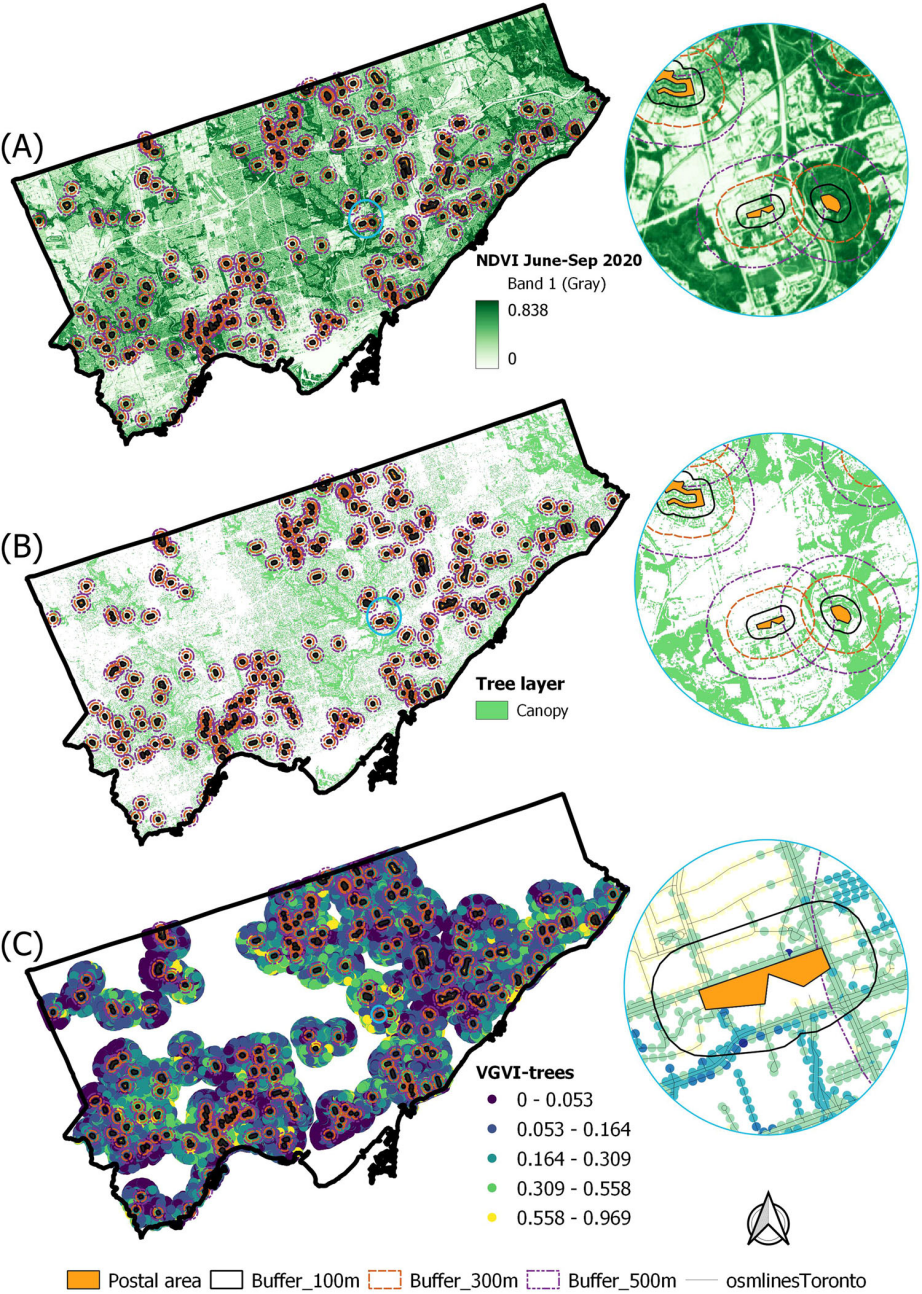
Neighbourhood greenness at sample locations. Spatial distribution of neighbourhoods and different greenness measures at multiple spatial scales (i.e., buffer sizes, 100, 300, and 500 m), indicating measures for **A** NDVI, **B** Canopy Cover, **C** VGVI at sampled locations (*n* = 223).

We used the survey responses from the City of Toronto that had postal codes assigned to them (*n* = 223). The locations of these responses were geographically distributed across the city, representing heterogeneous greenness conditions (Fig. 1). On average, respondents were somewhat satisfied with their trees (*M* = 3.68, SD = 0.72, on a 1–5 level of satisfaction scale), and slightly less satisfied with the management of these trees (*M* = 3.23, SD = 0.84, on a 1–5 level of satisfaction scale) (details in Supplementary (Tables 3–4). Also on average and using the middle buffer size around the postal code of the respondent, or 300 m, respondents had a relatively moderate NDVI (*M* = 0.36, SD = 0.10), moderate percentage tree canopy cover (*M* = 0.26, SD = 0.11), and low VGVI (*M* = 0.16, SD = 0.07), based on what is commonly found for these measures in urban areas^1,30–34^ (details in Supplementary Table 6).

### Associations of greenness measures and satisfaction measures

**Fig. 2:**
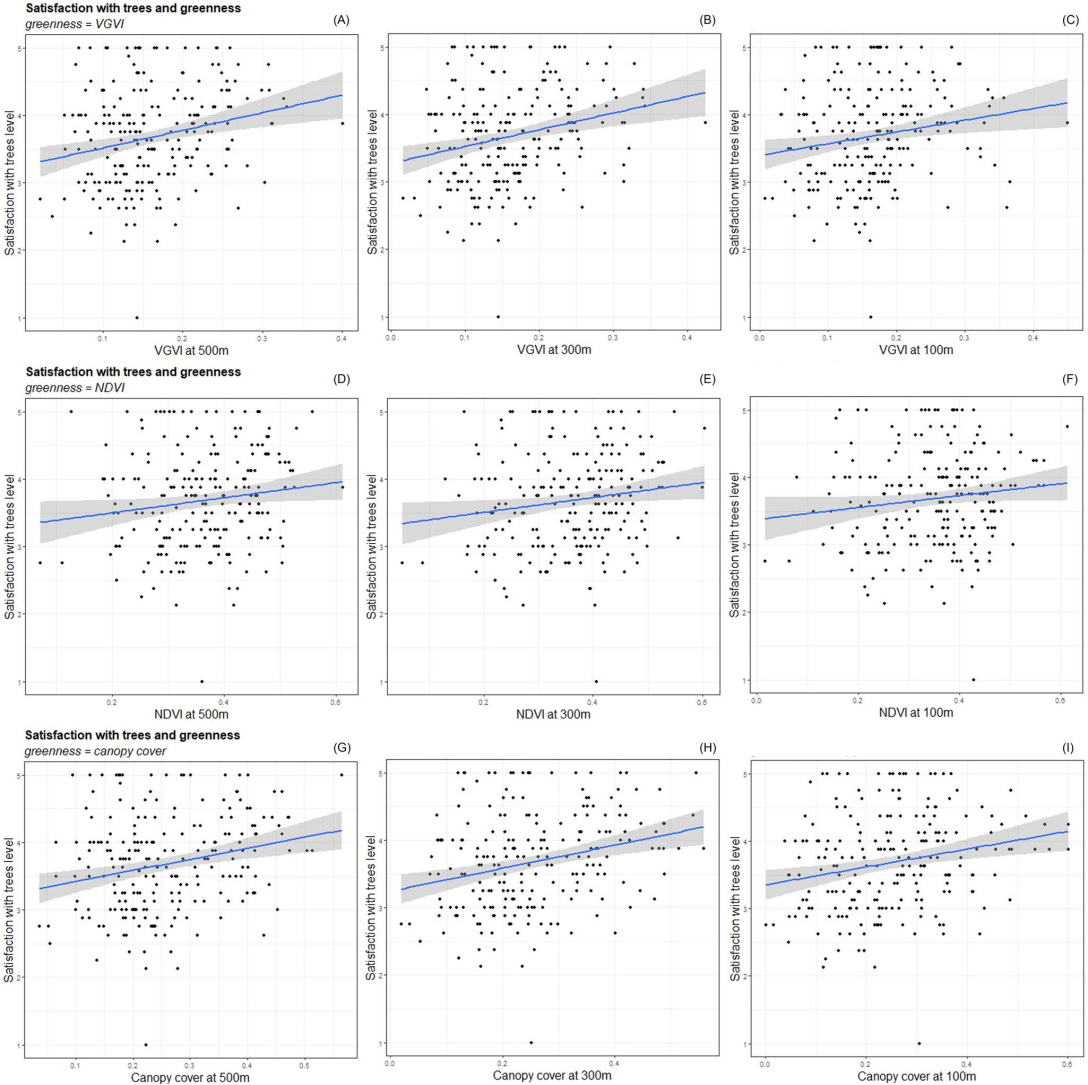
Associations of satisfaction urban trees and neighbourhood greenness. Scatter plots and linear trend lines with 95% confidence intervals showing the associations between satisfaction with urban trees and three mean neighbourhood-level greenness measures: **A** VGVI at 500 m, **B** VGVI at 300 m, **C** VGVI at 100 m, **D** NDVI at 500 m, **E** NDVI at 300 m, **F** NDVI at 100 m, **G** canopy cover at 500 m, **H** canopy cover at 300 m, and **I** canopy cover at 100 m, in the City of Toronto based on survey data (*n* = 223).

Regarding the association between residents’ level of satisfaction with urban trees and the three neighbourhood-level greenness measures at different buffer sizes, we observed that, just visually, the scatter plots of these bivariate relationships did not show a clear pattern. However, in the GLM analyses we found that canopy cover and VGVI had a positive association, when controlling for cognitive, social-ecological context, and demographic factors (Fig. 2, Table 1). In addition, we observed consistently comparatively stronger effect size (coefficient values) for VGVI than canopy cover, indicating comparatively stronger associations of VGVI with satisfaction than canopy cover. The relationships were not significant for NDVI.

**Table 1:** Generalized linear models for listed variables and their association with the level of satisfaction with urban trees controlling for cognitive, social-ecological context, and demographic factors in Toronto, Canada, indicating statistically significant values in bold.

Modelled variable	Estimate	95% confidence interval (CI) (lower, higher)	Significance (*p*-value)		Null deviance (D)	Residual deviance (D)	AIC
VGVI at 500 m	**2.50**	**(0.93, 4.08)**	**<0.01**	**	108.5	93.6	461.3
300 m	**2.46**	**(1.03, 3.88)**	**<0.001**	***	108.5	93.0	459.9
100 m	**1.66**	**(0.27, 3.05)**	**0.02**	*	108.5	96.2	467.0
NDVI at 500 m	0.92	(−0.16, 2.01)	0.10		108.5	97.7	470.3
300 m	0.97	(−0.04, 1.98)	0.06		108.5	97.6	470.1
100 m	0.79	(−0.20, 1.79)	0.12		108.5	98.3	471.7
Canopy cover at 500 m	**1.57**	**(0.61, 2.53)**	**<0.001**	***	108.5	93.4	460.92
300 m	**1.70**	**(0.80, 2.59)**	**<0.001**	***	108.5	92.2	458.1
100 m	**1.37**	**(0.51, 2.24)**	**<0.01**	**	108.5	94.8	464.0

*n* = 223.

Each variable is modelled independently at the individual response level. The models control for the following: level of nature relatedness (NR6); level of knowledge of trees; tree in front of home; years in neighbourhood; age (median); Canadian born; English-as-Second-Language; owns a house; education: university degree; ethnicity: white; gender: female; and belongs to an environmental organization (details in Supplement 4).

Estimated effects are nondirectional, standard two-tailed hypothesis testing, with a two-sided confidence interval.

Significance codes: <0.001***, <0.01**, <0.05*.

In relation to the association between residents’ level of satisfaction with the management of urban trees and the three neighbourhood-level greenness measures at different buffer sizes, we did not find any significant associations (results in Supplementary Fig. 9 and Supplementary Table 7).

With regards to the magnitude or strength of the association at different neighbourhood sizes, we found that the association between urban tree satisfaction and canopy cover and VGVI existed at all buffer sizes (i.e., 100, 300, and 500 m). However, the association was strongest (i.e., higher correlation and coefficient values) at 300 and 500 m for both measures, in that order (Table 1).

## Discussion

This study examined the relationship between neighbourhood-level urban greenness measures and people’s satisfaction with urban trees and their management to begin to address the gap in the understanding of how people perceptually respond to the abundance and visibility of urban trees. In our analysis of this relationship, we controlled for cognitive, demographic, and social-ecological factors that have been shown to influence perceptions of urban trees, such as knowledge of trees, gender, age, education, among other factors. This allowed us to better examine the relationship between the abundance of specific ecological structures and the subjective level of satisfaction with these structures, with a focus on the trees that people experience in their day-to-day life (i.e., trees in their neighbourhoods). Previous research has highlighted that personal identity plays an important role in people’s perceptions of urban trees, including preferences for urban tree form, benefits associated with urban trees, or attitudes associated with urban tree planting^15–18,27–29^. In this context, our results add a better understanding of how people’s daily experiences with trees also shape these perceptions while controlling for various personal identity factors. This way we can move towards a more complete model of people’s perceptions of urban nature and the role of cognitive, identity, and experiential factors.

As hypothesized, satisfaction with trees had the weakest relationship with NDVI, a measure that captures all vegetation. Previous research shows how higher NDVI values could indicate both more trees and few trees but very extensive ground-level vegetation^39^. The comparatively stronger relationship for VGVI than for neighbourhood canopy cover found in this study demonstrates the higher relevancy of VGVI in capturing the abundance of trees people would see when walking around their neighbourhood. Canopy cover is limited to a two-dimensional, “bird’s eye” measure of greenness. While many studies rely on canopy cover to represent urban forest presence, our results suggest that this measure does not adequately capture how people are experiencing urban trees. Particularly in urban environments with a varying density of buildings that may block views of trees, canopy cover may overestimate how people’s ability to see trees in their neighbourhood. The eye-level tree visibility analysis using VGVI might better capture what people see in their neighbourhood, therefore being more reflective of their experiences with trees that shape perceptions like satisfaction. Also, as hypothesized, the associations between satisfaction and the greenness measures were comparatively stronger at larger spatial scales possibly due to the presence of spatial aggregation effects associated with MAUP (details in Methods). In our case, at larger spatial scale we might have more trees, and more aggregation effect^30,38^. In addition, we argue that at larger spatial scale (e.g., 500 m), day to day experience of trees might be better reflected due to daily mobility within the neighbourhood for different activities compared to smaller scale (e.g., 100 m), which might only represent the immediate surrounding of people’s home. Therefore, urban tree management should consider the visibility or abundance of trees within a scale that might better represent peoples’ day-to-day experience of trees for greater satisfaction with trees.

Given its positive relationship with urban tree abundance and visibility, satisfaction with urban trees may play an important role as a useful measure of the difference between people’s expectation of their urban trees and how they cognitively process their actual experience of these trees^16–18^. While there are many studies that have examined universal preferences of the urban environment with the aim of integrating nature into this environment in broad terms^13,14^, there are still few studies that study how people perceptually respond to specific ecological structures, including, for example, people’s attitudes or preferences associated with the abundance, diversity, arrangement, visibility, or condition of urban trees^15,18^. A better understanding of whether communities are satisfied with their urban trees can lead to better informed urban tree management decisions^19^. This understanding can also support monitoring of the success of urban-tree enhancement efforts and help address existing inequities in the distribution of urban trees in the urban social landscape^8^.

The fact that we did not find any association between greenness measures and satisfaction with the management of urban trees deserves some explanation. Satisfaction with urban tree management may reflect more abstract, as well as more established and less easy to change perceptions of people’s experience with local municipal governments and their decisions about urban trees^18^. While this measure may be useful to assess how people feel satisfied with the decisions of the city about urban trees, it may not be associated with people’s daily experiences with urban trees. Rather, it may reflect more complex community dynamics such as, for example, people’s expectations of urban tree stewardship and governance^12^. Further research should explore the cognitive, social-ecological context, and demographic factors that may lead to higher or lower satisfaction with the management of urban trees.

## Methods

### Ethics statement

The protocol of this study was approved by the University of Toronto Ethic Review Board, with Ethic Protocol No. 00040945. Informed consent was obtained from all participants by describing the study in an online plain language statement of the research to all participants and by participants acknowledging their consent when choosing to continue answering the survey.

### Theoretical framework

This research was based on the cognitive hierarchy model, which differentiates perception responses in terms of the constructs of values, beliefs, attitudes, and preferences, among others, and organizes these constructs hierarchically according to their level of abstraction, ease of or resistance to change, and number, among others^40,41^ (details in Supplementary note 1 with additional references in Supplementary references). Based on this model, we conceptualized satisfaction as a relatively specific and less abstract perception response. We also theorized that satisfaction with urban trees may be closely linked with the urban trees people experience daily.

### Context

We designed and delivered a survey in the City of Toronto (Ontario, Canada). The survey was part of a larger research program regarding people’s perceptions of urban trees across Canadian cities. The City of Toronto is the largest in Canada with 2,794,356 people (https://www150.statcan.gc.ca/t1/tbl1/en/tv.action?pid=9810000101). The city is located in Southern Canada, in the Great Lakes region, which has a moderate humid continental climate (Köppen classification Dfa) (https://www.climate.weather.gc.ca). The city includes high-density neighbourhoods with high-rise, multi-family dwellings, moderate-density neighbourhoods with semi-detached and detached housing on small lots, as well as more suburban-style single-family dwellings on larger lots. The area is one of the most culturally diverse in North America, with 51.2% of residents identifying as visible minority, higher than the Canada-wide value of 22.3% (https://www12.statcan.gc.ca/census-recensement/2021/ref/98-500/006/98-500-x2021006-eng.cfm). The city has developed an ambitious agenda to increase urban tree abundance and address inequities in urban tree distribution (http://www.toronto.ca/trees/). The canopy cover of the city, as calculated in 2013, was 28%, with a target to increase this to 40% without a specified timing (https://www.toronto.ca/wp-content/uploads/2017/12/8e0e-Strategic-Forest-Management-Plan-2012_22.pdf).

### Survey

The survey included questions about people’s perceptions of urban trees, using existing validated measures^15^ (details of measures in Supplementary methods 1). The measures of *satisfaction with urban trees* and *satisfaction with urban tree management* were each based on an 8-item scale, with each item rated by the degree of satisfaction in a 5-point scale. We also measured people’s *level of nature relatedness*^36^ using the NR6 scale^42^, level of knowledge of trees, having a tree in front of the home, and belonging to an environmental organization^37^ (details of measures’ properties in Supplementary Tables 2–5). We captured demographic data, including education, age (median), gender, years living in the neighbourhood, home ownership, and cultural diversity, such as born in Canada, English-as-Second-Language (ESL; excludes First Nation, Métis, and Aboriginal Canadian languages), and ethnicity (based on Statistics Canada; see https://www150.statcan.gc.ca/t1/tbl1/en/ tv.action?pid=9810000101; https://www12.statcan.gc.ca/census-recensement/2021/ref/98-500/006/98-500-x2021006-eng.cfm). Sharing postal codes was voluntary in the survey (details of demographics in Supplementary Table 1).

The delivery of the survey was based on a systematic, random, and probabilistic sampling approach^43^. We used an electronic online panel survey, an internet-based, self-administered data collection technique that is validated by sociodemographic parameters given that it uses an established panel of respondents. It has been used before in urban tree public opinion research^29^, so there are well established procedures. We used the panel managed by Asking Canadians® (www.askingcanadians.com), which has access to more than 1 million panellists in Canada. In addition to the sociodemographic validations, we developed a protocol to ensure geographic representativeness (details in Supplementary methods 2).

### Greenness assessment

We measured greenness abundance and visibility by calculating NDVI, tree canopy coverage (%), and VGVI for trees, using Euclidean (e.g., straight line distance) buffer zones (i.e., 100, 300, and 500 m) around the postcode boundary of the respondents’ residential locations. These spatial extents are much smaller than those in previous studies (e.g., 1–3 km^44^), and are assumed to represent a walkable distance based on other studies (e.g., 400 m–1.6 km^31^).

In addition, we considered multiple buffer distances to account for two issues. First, the modifiable areal unit problem (MAUP), a source of statistical bias in spatial data analysis^45^. MAUP indicates that, as we aggregate spatial data into arbitrary spatial scales or zones, the spatial extent and size aggregation area influence the statistical relationship we observe^38,45^. In particular, when using buffer distance to aggregate greenness metrics, greater spatial aggregation may result in a more significant or stronger association with the outcome variables as the buffer distances become larger^46^. We selected smaller (i.e., 100 m) and larger buffers (i.e., 500 m) to test such sensitivity and ensure a more careful consideration of the spatial effect in the observed relationships. Second, we wanted to better represent people’s day to day experience with trees through multiple buffers. In this case, a 100 m buffer might capture the interactions with trees immediately adjacent to someone’s home. However, people move beyond their homes for everyday activities outside of their immediate home environment^47^. Day to day experiences with trees are shaped by more than the trees immediately adjacent to the home. So, these experiences may be better captured at larger buffer distance. We carefully selected 500 m as the largest buffer because this represents a reasonable walkable distance people might consider as neighbourhoods, and yet not too large to over-aggregate the greenness data. Analysing greenness data even at even larger buffers (e.g., 1600 m; see ref. ^44^) might over-aggregate the data, reduce variability in exposure values, and induce uncertainty in statistical models as noted by^46^.

We created the NDVI layer from Sentinel-2 satellite images obtained between June to September 2020. The period was chosen to reflect the phenological pattern and summertime vegetation conditions in Toronto. Sentinel-2 images (10 m) were selected over traditional Landsat (30 m) and MODIS (250 m) satellite images due to their relatively higher spatial resolution, and previous studies indicated Sentinel-2 images might be better at identifying urban greenery than Landsat and MODIS^10,48^. Google earth engine was used to search and select all the images for the summer period. A composite image was created by combining the median value for the best pixels identified for all the images of this period with minimum cloud cover (at least <10%). NDVI was calculated with the formula by^49^ using the RED (central wavelength 664.6 nm) and the near-infrared band (central wavelength 832.8 nm) of the Sentinel-2 images. NDVI values range between −1 to +1, where −1 indicates water and near 0 values indicate buildings or bare land; values about 0.2 indicate vegetation coverage, and higher values indicate denser forest coverage. We removed values below 0 to ensure we only consider possible image pixels that might contain vegetation. We estimated the mean NDVI value for each buffer zone for all the selected postcodes; hence we obtained average NDVI values at 100, 300, and 500 m.

Since NDVI cannot differentiate between vegetation types, such as grass and trees^39^, we measured tree abundance by estimating the canopy percentage within the buffer zones using a high-resolution (2 m) land cover dataset from the “Automated Land Cover Analysis-2018 Tree Canopy Study” created by the City of Toronto. Out of eight land cover types in the land cover data, the “tree” class was extracted in QGIS (v20). Further details and tree canopy data can be found at: https://ckan0.cf.opendata.inter.prod-toronto.ca/tl/dataset/forest-and-land-cover. Once extracted, we estimated the percentage of the area within the buffer zones (i.e., 100, 300, and 500 m) for each postcode covered with tree canopy.

Since canopy cover is a top-down (e.g., bird’s eye view) measure of tree abundance in two-dimensional space, we also measured how people viewed trees at eye level in a three-dimensional space. For this we estimated the eye-level tree visibility using Viewshed Greenness Visibility Index (VGVI)^33^. Full details of the modelling process are provided in this reference. Instead of all greenery types, we only measured the visibility for trees using the VGVI index. We used high-resolution (2 m) digital elevation data from Ontario Digital Surface Model (details available at: https://geohub.lio.gov.on.ca/maps/mnrf::ontario-digital-surface-model-lidar-derived/about), along with canopy coverage layer to estimate VGVI for 300 m viewing distance at 20 m interval on the streets within the buffer zones. The viewing distance of 300 m was selected based on the argument that in urban contexts, green visibility might be better represented with a relatively small viewing distance^50^. Furthermore, we sampled street-only locations using OpenStreetMap data for Toronto to ensure we measure potential tree visibility around the home and within the neighbourhood activity areas. Previous studies also used street-level viewpoints in urban settings to estimate green visibility^34^. For all the sample points for each buffer zones, we estimated VGVI values using GVI R package (v 1.1) (available at 10.5281/zenodo.7057132) and calculated mean VGVI for each postcode area (details in Supplementary Figs. 6–8).

### Data analysis

We used R v. 4.2.1 (https://www.r-project.org/) to perform all the statistical analyses and to generate visualizations from the data. We used confirmatory factor analysis and reliability measures to verify the structure of the scales^18^. For the first, we used the *cfa* function in the *lavaan* R package (v. 0.6-9). For the second, we used the *alpha* function from the *psych* R package (v. 1.9) (α values are given in Supplementary Tables 2–5). For simplicity, we used the average indexes of the scales. To answer the research questions and associating satisfaction measures with greenness measures, we used regression-based analyses based on generalised linear models (GLM). We used the *glm* function with Gaussian error distribution in R. We ran individual models for each greenness measure (NDVI, canopy cover %, and VGVI) at each buffer zone (100, 300, 500 m) to predict the level of satisfaction with urban trees and the level of satisfaction with the management of urban trees at the individual response level (i.e., 18 GLMs overall). In all models we controlled for cognitive, social-ecological context, and demographic factors (details in Table 1) and 95% confidence intervals were calculated using the predict function. The distribution of residuals was checked for normality to confirm the assumptions of the model using variance inflation factors (VIFs) as well as residual plots^51^.

### Limitations

This research has used an approach to associate urban tree abundance and visibility with people’s perception responses to urban trees, in this case, people’s level of satisfaction with urban trees and their management. We acknowledge that the database of 223 residents is small, as we were limited by the lack of responses with 6-digit postal codes, as well as the availability of greenness data, which is currently only available for the City of Toronto and not for other Greater Toronto Area (GTA) municipalities. We recognize we did not account for neighbourhood characteristics (e.g., socio-economic disadvantage^31^), interactive effects (e.g., demographic age groups^44^), or levels of urbanity (e.g., urban typologies in an urban-rural gradient^21,44^). One reason for this was that the data and the scale of analysis were at the individual response level. Accounting for these factors would have involved averaging responses at the neighbourhood level, which would have been incongruent. Another reason already mentioned was that greenness data were only available for Toronto, though we had survey data for other municipalities in the GTA. Moreover, while we found an association between subjective community satisfaction and objective greenness measures, further exploration of these relationships in different contexts is warranted, particularly involving a wider range of multivariate data expressing cognitive, social-ecological, and demographic context factors, such as those considered in this study. A strength was that our sample of responses covered a wide range of cognitive, social-ecological context, and demographic characteristics and locations within the City of Toronto. We dealt with a very rich dataset that allowed us to account for the influence of more of these types of factors than most other studies. Nonetheless, further exploring these associations in different contexts, with bigger datasets, exploring different types of associative functions instead of just linear relationships (e.g., polynomial), and refining analytical techniques, can further advance research in this space.

### Reporting summary

Further information on research design is available in the Nature Research Reporting Summary linked to this article.

### Supplementary information

Supplemental material

Reporting Summary

## Data Availability

The remote sensing data, including base files, shape files, and modelling techniques, used and/or analysed during the current study (i.e., greenness measures) are available publicly and online, included in this published article and its supplementary information files, and/or available from the corresponding author on reasonable request. The social datasets generated and/or analysed during the current study (i.e., survey responses) are not publicly available due to restrictions imposed by the institutional ethics review board, as study participants did not consent to sharing this information. These social datasets are available from the corresponding author on reasonable request without any personal, temporal, or locational information to ensure confidentiality and anonymity of the research participants. Nonetheless, enough details about these datasets are included in this published article in aggregated form in the supplementary material.
