# Supplementary material for: Satisfaction with urban trees associates with tree canopy cover and tree visibility around the home
Source: NPJ Urban Sustain. 2023 Jun 23;3(1):37. doi: 10.1038/s42949-023-00119-8 (PMC11041773; doi:10.1038/s42949-023-00119-8)
Supplement: Supplementary file 1 — Supplemental material [file 42949_2023_119_MOESM1_ESM.pdf]

SATISFACTION WITH URBAN TREES ASSOCIATES WITH TREE CANOPY COVER AND TREE VISIBILITY AROUND THE HOME

Supplementary material

Contents

Supplementary note 1 .... *2*

Supplementary methods 1 .... *3*

Supplementary methods 2 .... *5*

Supplementary figures *7*

Supplementary tables *16*

Supplementary references *21*

Supplementary note 1

Cognitive hierarchy model

People perceive things in many ways, so there are different ways of measuring perception. A useful way of understanding perception is through the cognitive hierarchy model, which differentiates the cognitive constructs of values, beliefs, attitudes, norms, and preferences, among other commonly used terms in various disciplines – particularly in the context of the environment and natural resources –, including psychology, sociology, and human geography[1] . The model organizes perception responses according to their level of abstraction, ease of or resistance to change, and number, among other characteristics[2,3]. Lower level constructs, such as values – what is important to people or what guides people's decisions[4-6] – and beliefs – what people accept as true, including the consequence of doing something or how something should be[7,8] –, are thought to influence higher level constructs, such as attitudes – people's judgements or disposition towards things[9] – and preferences – how much people like things[10] (see *Supplementary figure 1*). The model may have various variations, depending on the cognitive pathway being tested, including values-beliefs-attitudes (VBA), or values-beliefs-norms (VBN), among others. Attitudes, such as level of satisfaction with urban trees, may be theoretically easier to change with a person's immediate experience, such as the number of urban trees around the person, but it may also depend cognitively on a person's values, beliefs, among other perceptions.

Supplementary methods 1

Survey questions

Order of questions does not reflect the actual order in the survey

No.	*Question*	*Items*	*Code*	*Measure*
1	*Are you over 18 years old?*			Yes/No
2	*In which city boundary or municipality do you live in?* 1) Brock, 2) Clarington, 3) East Gwillimbury, 4) Georgina, 5) Halton hills, 6) Markham, 7) Milton, 8) Mississauga, 9) New Market-Aurora, 10) Scugog, 11) Toronto, 12) Whitby, 13) other			Selection (strict) (includes dummy cities)
3	*Please rate your knowledge of...*	1. Planting a tree	k.1.plant	1-5 level of knowledge, with 1 very low and 5 very high
		2. Caring for a tree	k.2.care	
		3. Identifying trees native to your area	k.3.identify	
		4. Knowing the names of everyday trees	k.4.know.name	
4	*Do you have a tree planted in front of your home (house/residence/building)?*			Yes/No/ Don't know / Prefer not to answer
5	*Have you ever contacted your city council to prune, cut, or plant trees?*			
6	*How satisfied are you with the following things about the trees in your neighbourhood?*	1. Suitability (right tree for the location)	s.t.1.suitable	1-5 level of satisfaction, with 1 Not satisfied at all and 5 Extremely satisfied
		2. Attractiveness (looks good)	s.t.2.attractive	
		3. Safety (low risk of damage or harm)	s.t.3.safe	
		4. Quantity (right number of trees for area)	s.t.4.abundant	
		5. Nativeness (Canadian trees)	s.t.5.native	
		6. Exoticness (trees from overseas)	s.t.6.exotic	
		7. Diversity (many kinds of trees)	s.t.7.diverse	
		8. Habitat (trees that provide shelter or food for animals)	s.t.8.habitat	
7	*How satisfied are you with the following things about the management of trees in your neighbourhood?*	1. Timely replacement of trees that have been removed	s.m.1.timely	
		2. Removal of unhealthy or dead trees	s.m.2.remove unhealthy	
		3. Equitable planting of trees, across all suburbs so everybody has a tree near them	s.m.3.equitable	
		4. Responsiveness to people's requests to prune, remove, replace, or plant trees	s.m.4.responsive to requests	
		5. Investment in tree planting and caring	s.m.5.invest	
		6. Engagement with the community on decisions related to public trees	s.m.6.engage	
		7. Appropriate management of living trees (pruning, watering, etc.)	s.m.7.maintenance	
		8. Appropriate management of living trees to provide habitat for wildlife (hollows, etc.)	s.m.8.habitat	

No.	*Question*	1. *Items*	*Code*	*Measure*
8	*Please rate the extent to which you agree with each statement. Please respond as you really feel, rather than how you think "most people" feel:*	My ideal vacation spot would be a remote, wilderness area	nr6.1	1-5 level of agreement, with 1 strongly disagree and 5 strongly agree
		I always think about how my actions affect the environment	nr6.2	
		My connection to nature and the environment is a part of my spirituality	nr6.3	
		I take notice of wildlife wherever I am	nr6.4	
		My relationship to nature is an important part of who I am	nr6.5	
		I feel very connected to all living things and the earth	nr6.6	
9	*How long have you lived in your current neighbourhood? Please type number of years*			[open-ended numeric]<br>Don't know / Prefer not to answer
10	*What is your housing situation?*<br>Options: 1) I rent, 2) I own my own home or am in the process of doing so, 3) Other			Selection (strict)<br>Don't know / Prefer not to answer
11	*In which decade were you born?*<br>Options: 1) 1920s, 2) 30s, 3) 40s, 4) 50s, 5) 60s, 6) 70s, 7) 80s, 9)2000s,			
12	Where you born in Canada?			Yes/No / Don't know / Prefer not to answer
13	*Do you grow up speaking any other language other than English or do you speak any other language other than English with your family/at home?*			Yes/No / Don't know / Prefer not to answer<br>[open-ended text]
14	*What is your highest educational degree awarded?*<br>Options: 1) did not complete high school; 2) high school / secondary school, 3) technical diploma/certificate, 4) Bachelors or undergraduate university degree, 5) Graduate diploma/certificate, 6) Doctorate/PhD university degree			Selection (strict)<br>Don't know / Prefer not to answer
15	*Do you belong to an environmental organization?*			Yes/No / Don't know / Prefer not to answer
16	*Do you identify as an Indigenous, Métis, or Aboriginal Canadian?*			
17	*What gender do you identify with?*<br>Options: Male, Female Other [Define if you want]			Selection (strict)<br>Don't know / Prefer not to answer

Supplementary methods 2

Survey delivery protocol

We designed and delivered our electronic online panel survey to be delivered across the metropolitan are of Toronto, or Greater Toronto Area (GTA). The survey delivery and respondent recruitment were based on two criteria: geographic representativeness, achieved by using an adapted urban gradient approach[11]; and demographic representativeness, achieved by using an established panel for sourcing our responses.

Geographic representativeness in our study meant sourcing responses from a representative sample of different types of urban areas within a metropolitan area. The GTA spans urban areas of high development and population density (inner), to areas of low density and high population growth (outer), and areas in between (middle). We aimed to target all these types of urban areas by selecting specific municipalities within the GTA for survey delivery. To cover all these types of urban areas, we targeted specific municipal governments that met these criteria within the GTA following local guidelines of municipal classification (https://www.ontario.ca/document/ontario-municipal-councillors-guide/10-land-use-planning). However, municipal government boundaries in Canada have variable population size. Selected municipal governments in the GTA are not be comparable in terms of population size. Also, since we used an established panel for sourcing our responses (details below), there was a risk that not enough panellists existed within selected municipal governments, particularly within outer governments (examples below). Therefore, we ran the risk of not meeting our established response quotas.

We addressed these challenges in two ways. First, we joined or split selected municipal governments to achieve more comparable population size between selected areas and more comparable quotas. Second, we selected additional backup municipal governments from similar urban areas. We then delivered our survey to these selected municipal areas, which included the municipalities of: Toronto (classified as inner), Mississauga, Markham, Whitby (middle), Milton, Clarington, and East Gwillimbury (outer), and created stratified sampling quotas based on the municipal population size and a targeted sample size of 2,000 responses. We split the municipalities of Toronto, Mississauga, and Markham into randomly selected wards. We joined Milton with Halton Hills, Clarington with Brock and Scugog, and East Gwillimbury with Georgina and New Market-Aurora. This addressed the risk of not having enough panellist within outer urban areas (i.e., Milton, Halton Hills, Clarington, Brock, Scugog, East Gwillimbury, Georgina, New Market-Aurora) to meet established quotas. The additional municipalities served as backup municipalities. After selecting target areas, we extracted postal codes and forwarded these to the panel company to target the selected urban areas.

We used the panel company Asking Canadians® (www.askingcanadians.com) to deliver the survey and source responses. The company has access to more than 1 million participants, or panellists, in Canada. The respondents of the survey were self-selected and were compensated with a nominal fee reflecting the approximately ten minutes it took to complete. This fee is typical in electronic panel surveys (see references in main text) and has the goal of reducing self-selection bias. All stratified sampling quotas were either met or surpassed. The survey was delivered in the summer, May to June 2021.

The total targeted population, based on the population of the selected municipalities, was 1,997,134 people, or 34% of the GTA population. We received 2,015 valid responses for this

survey, of which 934, or 46% of the dataset, were from the City of Toronto. Also, out of the total dataset of 2,015, about 30% had full postal code information (i.e., 6-digit postal code). Of the dataset from the City of Toronto, we only obtained 223 responses with full postal codes. Based on this, the final useable number of respondents for this study was n = 223.

Supplementary figures

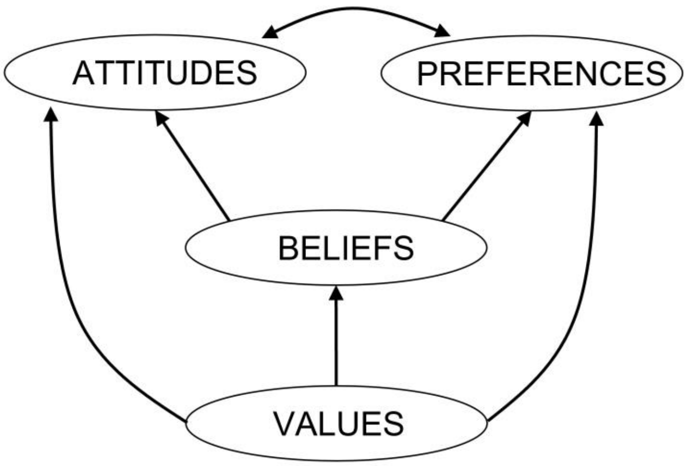

Supplementary figure 1: A simplified illustration of the cognitive hierarchy model used in this work (for an explanation, see *Supplementary note 1*)

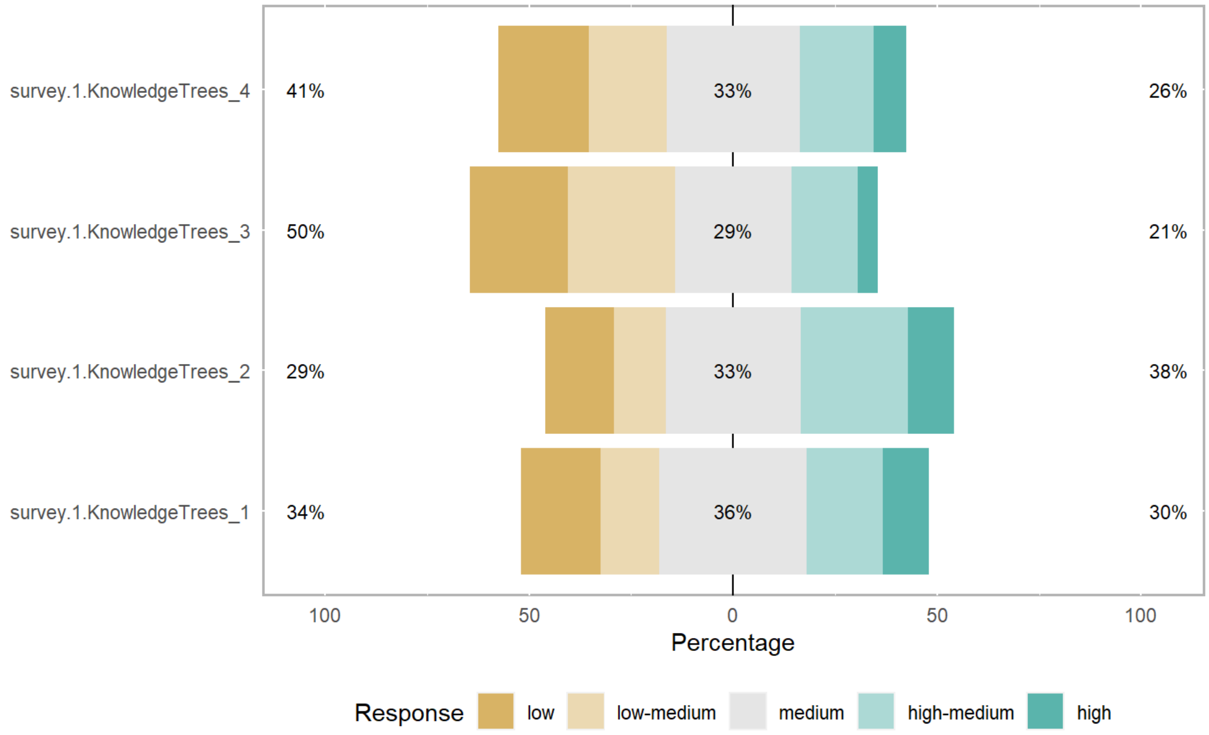

Supplementary figure 2: Likert-style plot for the Knowledge of trees scale, using data from the online panel survey in Toronto, Canada, n=223 (see also *Supplementary table 2*)

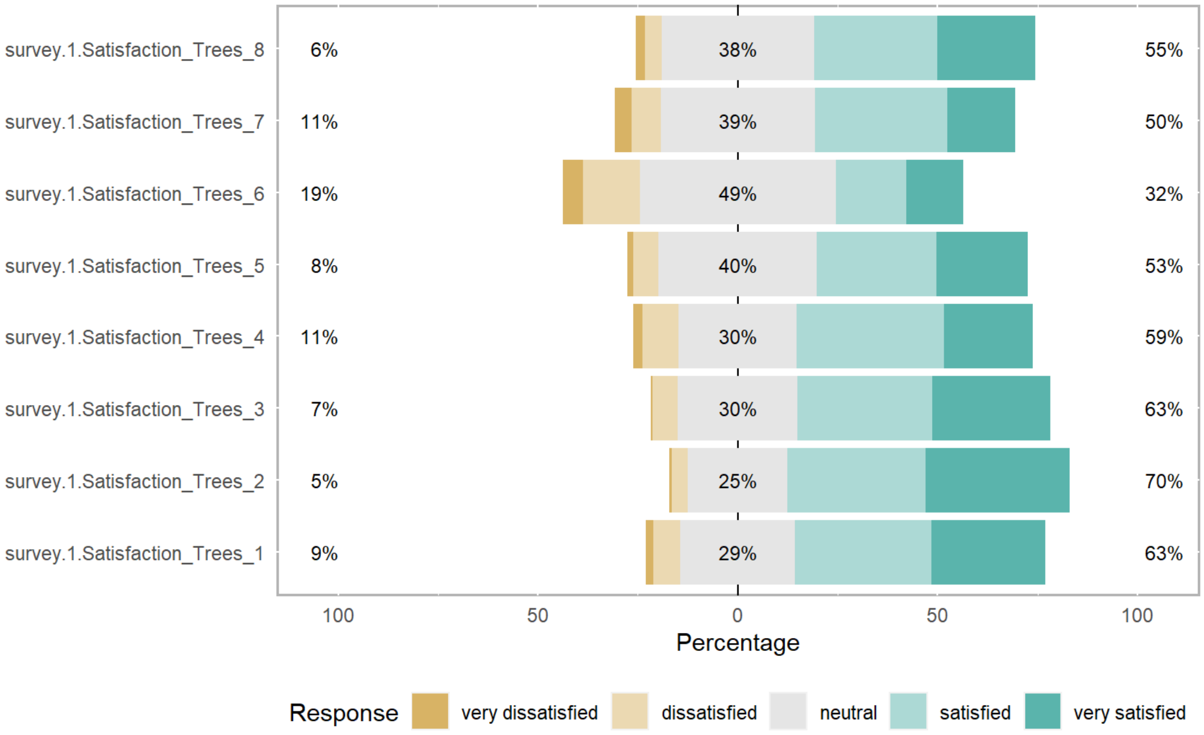

Supplementary figure 3: Likert-style plot for the Satisfaction with urban trees scale, using data from the online panel survey in Toronto, Canada, n=223 (see also *Supplementary table 3*)

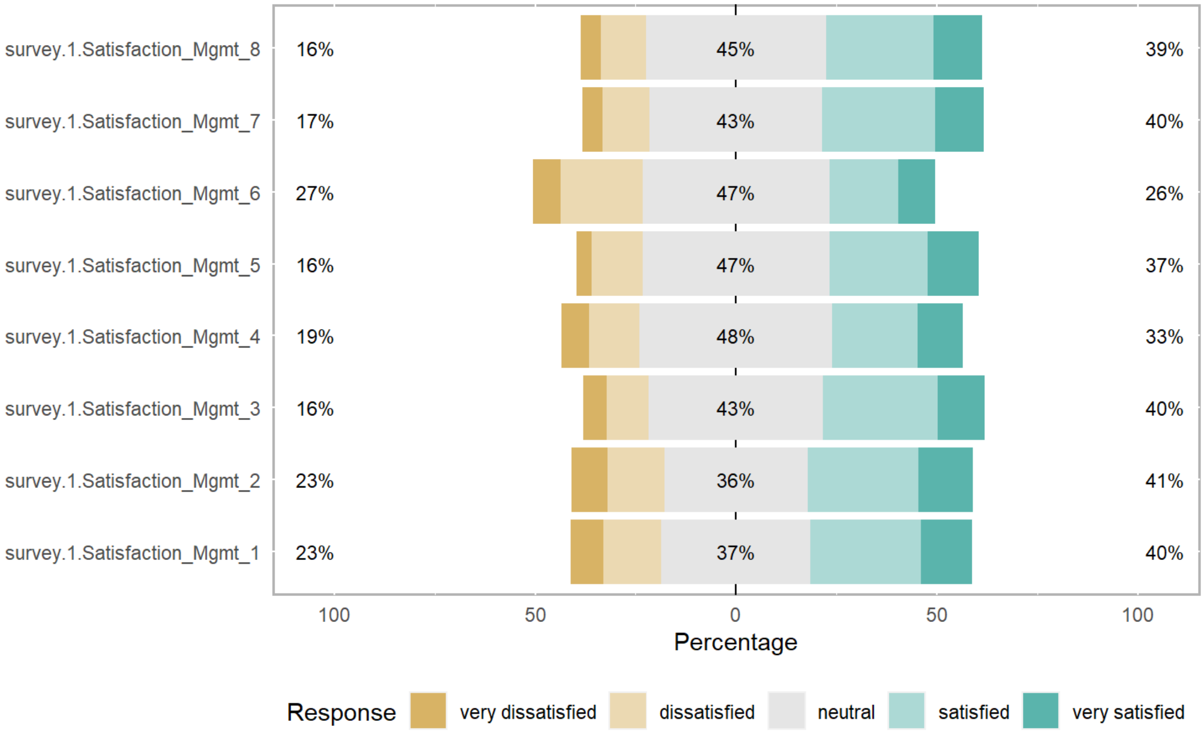

Supplementary figure 4: Likert-style plot for the Satisfaction with the management of urban trees scale, using data from the online panel survey in Toronto, Canada, n=223 (see also *Supplementary table 4*)

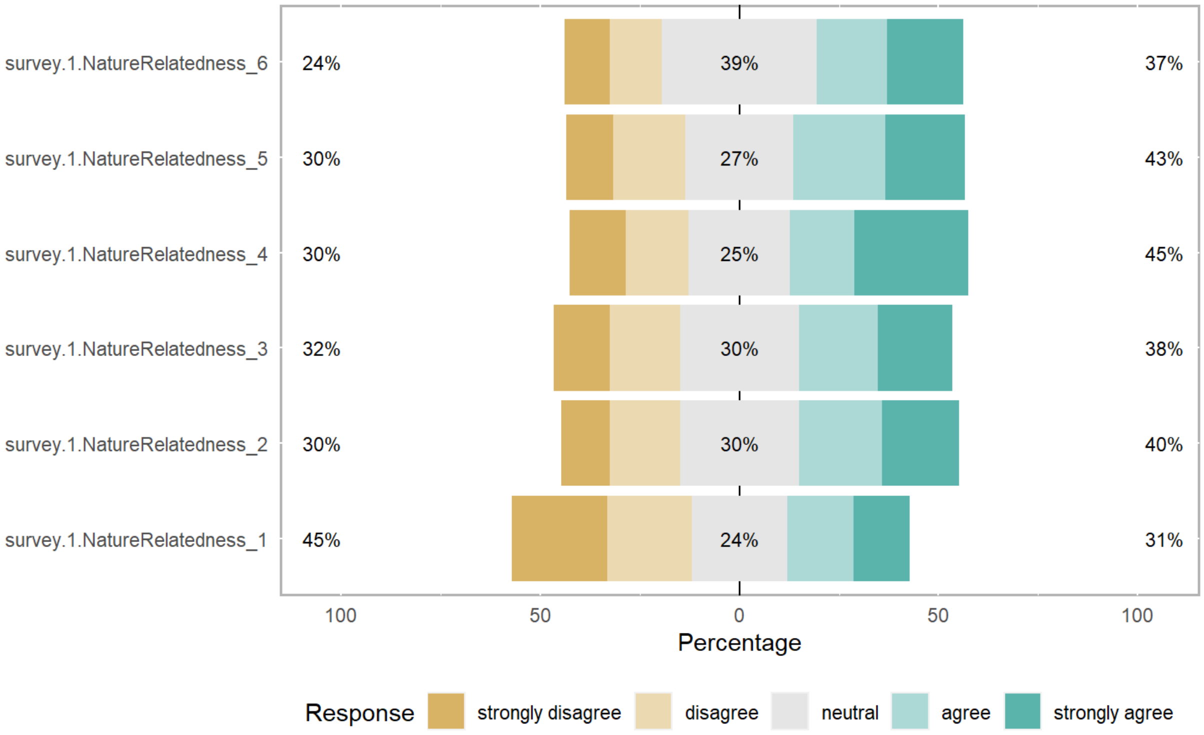

Supplementary figure 5: Likert-style plot for the Nature Relatedness (NR6) scale, using data from the online panel survey in Toronto, Canada, n=223 (see also *Supplementary table 5*)

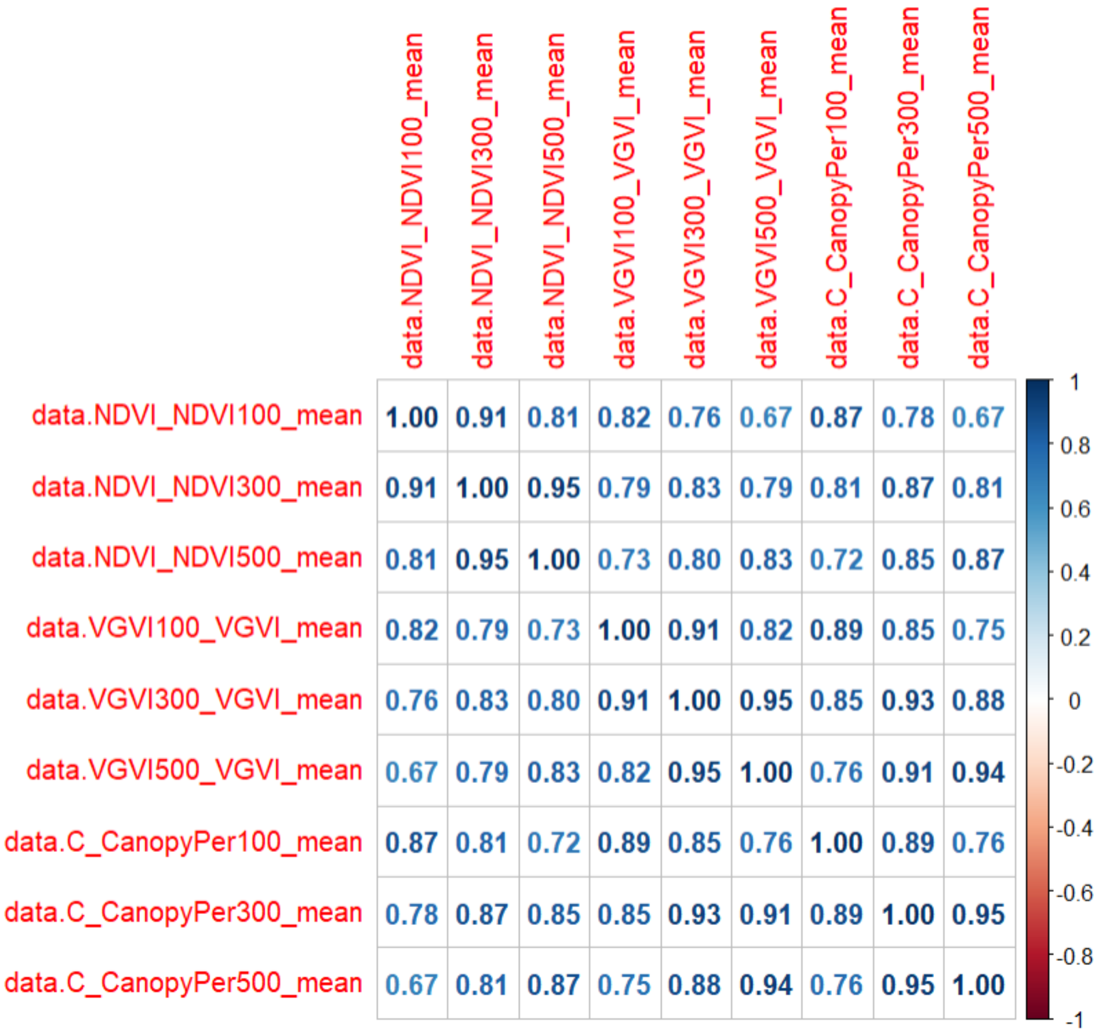

Supplementary figure 6: Correlation plot showing Pearson correlation coefficients among greenness measures, including NDVI, VGVI, and canopy cover, using data from the online panel survey in Toronto, Canada, n=223 (see main text, *Methods*)

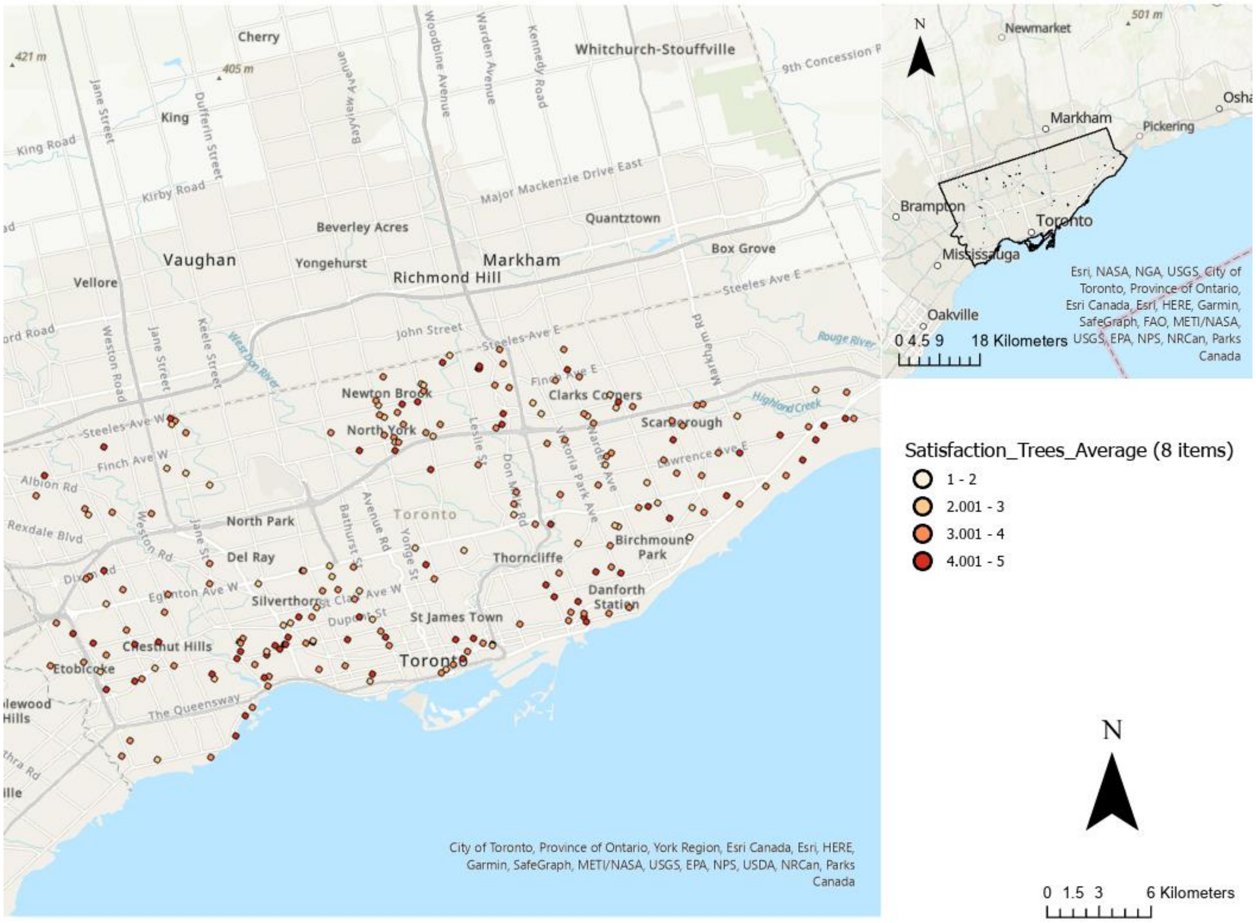

Supplementary figure 7: Map indicating locations of survey responses and values for the Satisfaction with urban trees scale, using data from the online panel survey in Toronto, Canada, n=223 (see main text, *Methods*)

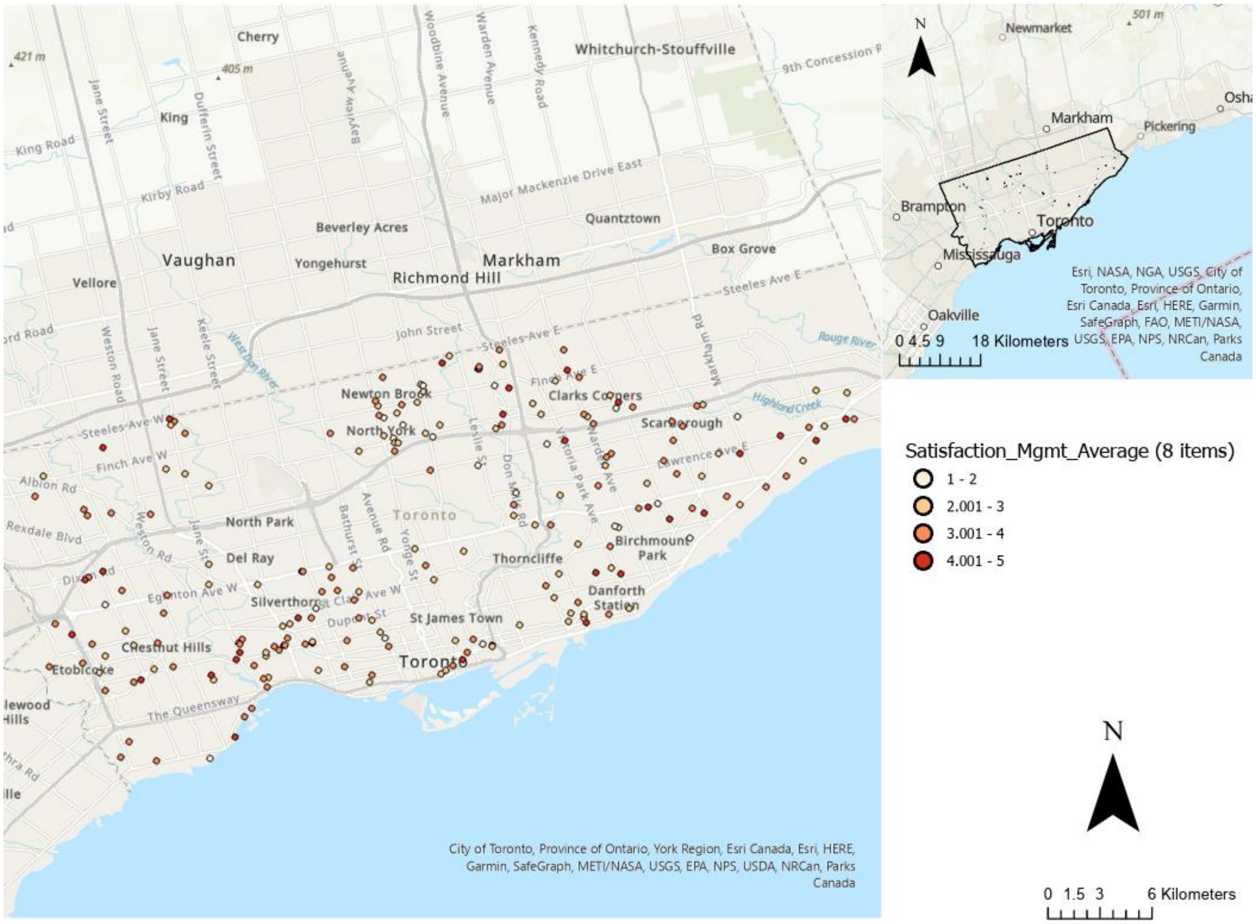

Supplementary figure 8: Map indicating locations of survey responses and values for the Satisfaction with the managmeent of urban trees scale, using data from the online panel survey in Toronto, Canada, n=223 (see main text, *Methods*)

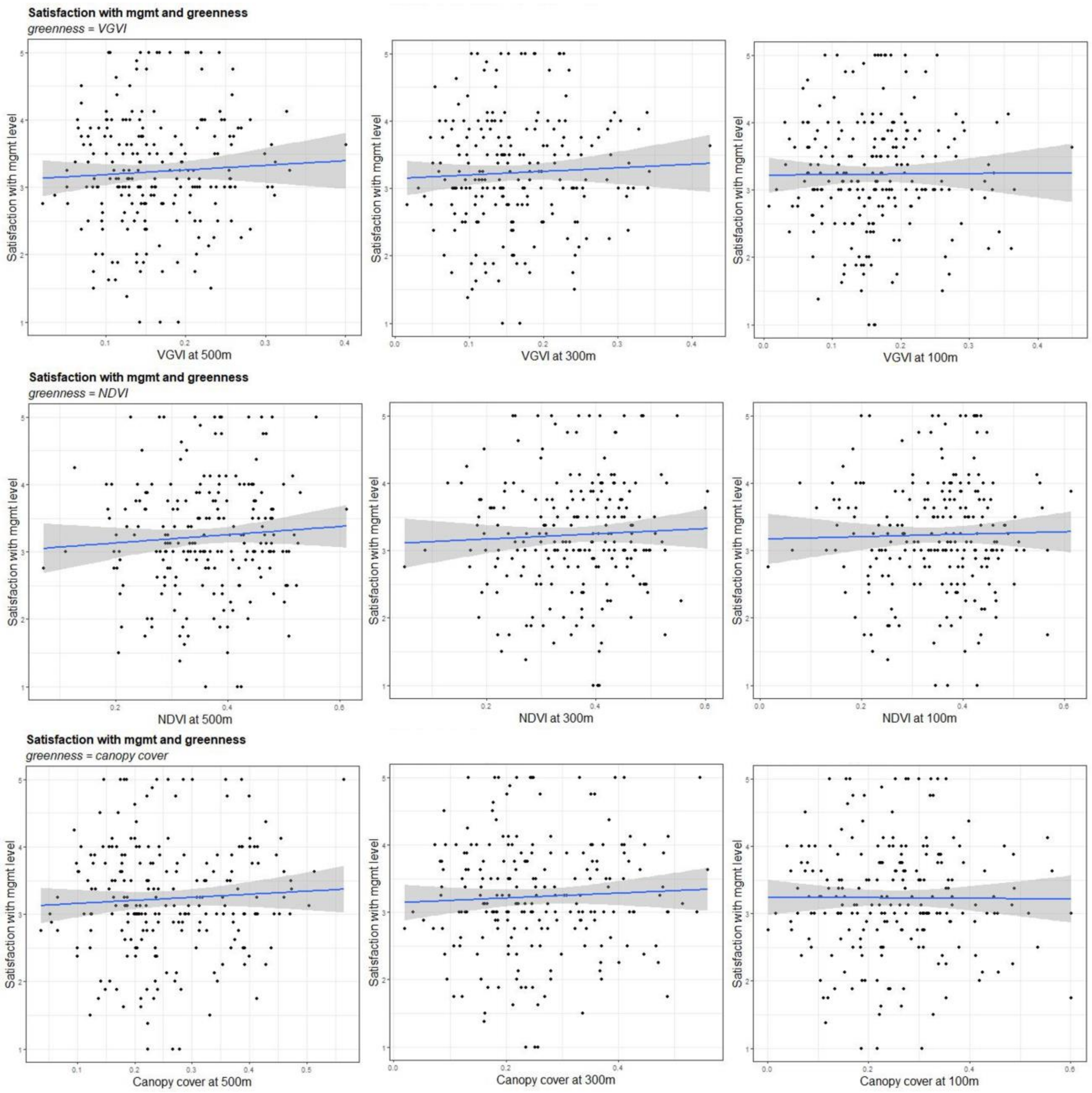

Supplementary figure 9: Scatter plots and linear trend lines with 95% confidence intervals showing the associations between satisfaction with the management of urban trees and three mean neighborhood-level greenness measures: VGVI, NDVI, and canopy cover, at 500m, 300m, and 100m, in the City of Toronto based on survey data (n=223).

Supplementary tables

Supplementary table 1: Demographic profile for the aggregated data from the online panel survey in Toronto, Canada

Factor						*Census data* [2]	
	Mean [1]	*SD*	*SE*	*Max*	*Min*	*Toronto (as mean or % of total)*	*Canada (as mean or % of total)*
City of Toronto yes =1 [2]	0.46	0.50	0.01	1.00	0.00	100%	7.6%
Tree in front of home yes=1	0.81	0.39	0.01	1.00	0.00	n.a.	n.a.
Years living in neighbourhood	15.97	12.53	0.29	86.00	0.00	n.a.	n.a.
Home own yes=1	0.70	0.46	0.01	1.00	0.00	52.8%	67.8%
Age (median)	48.74	16.77	0.38	95	18	39.9	41.1
Canadian born yes=1 [3]	0.65	0.48	0.01	1.00	0.00	51.0%	76.1%
English as second language (ESL) yes=1	0.34	0.47	0.01	1.00	0.00	49.1%	44.0%
Education level: university degree yes=1	0.65	0.48	0.01	1.00	0.00	23.3%	19.0%
Environmental organization yes=1	0.06	0.23	0.01	1.00	0.00	n.a.	n.a.
First Nations, Métis, and Inuit yes=1	0.01	0.09	0.00	1.00	0.00	1.3%	6.2%
Gender: Female yes=1	0.50	0.50	0.01	1.00	0.00	52%	50.4%
Ethnicity: White [4]	0.56	0.48	0.01	1.00	0.00	47.9%	57.1%

n=2,015
n.a. = no data available
1. The mean of a variable gives an indication on how many responses filled the stated condition. In cases when this condition was a binary choice between yes and no, or, conversely, as 1 and 0, then the number can be interpreted also as a %.
2. Source: https://www150.statcan.gc.ca/t1/tbl1/en/tv.action?pid=9810000101, retrieved May 2022
3. Source: https://www12.statcan.gc.ca/nhs-enm/2011/as-sa/99-010-x/99-010-x2011001-eng.cfm, retrieved May 2022
4. Source: https://www12.statcan.gc.ca/census-recensement/2021/ref/98-500/006/98-500-x2021006-eng.cfm, retrieved May 2022

Supplementary table 2: Statistical properties of the Knowledge of trees scale, using data from the online panel survey in Toronto, Canada, n=223 (see also *Supplementary figure 2*)

Factors and Items	*Names in graph*	*Mean*	*Standard Deviation (SD)*	*Standard Error (SE)*	*Reliability of all items (standardized Cronbach alpha)*
**as average index**		**2.78**	**1.08**	**0.02**	
k.1.plant	survey.1.KnowledgeTrees_1	2.93	1.26	0.03	
k.2.care	survey.1.KnowledgeTrees_2	3.02	1.20	0.03	0.91
k.3.identify	survey.1.KnowledgeTrees_3	2.48	1.20	0.03	
k.4.know.name	survey.1.KnowledgeTrees_4	2.70	1.25	0.03	

n=223

Supplementary table 3: Statistical properties of the Satisfaction with urban trees scale, using data from the online panel survey in Toronto, Canada, n=223 (see also *Supplementary figure 3*)

Factors and Items	*Names in graph*	*Mean*	*Standard Deviation (SD)*	*Standard Error (SE)*	*Reliability of all items (standardized Cronbach alpha)*
**as average index**		**3.68**	**0.72**	**0.02**	
s.t.1.suitable	survey.1.Satisfaction_Trees_1	3.73	0.94	0.02	
s.t.2.attractive	survey.1.Satisfaction_Trees_2	3.94	0.90	0.02	
s.t.3.safe	survey.1.Satisfaction_Trees_3	3.85	0.92	0.02	
s.t.4.abundant	survey.1.Satisfaction_Trees_4	3.65	1.01	0.02	0.90
s.t.5.native	survey.1.Satisfaction_Trees_5	3.66	0.93	0.02	
s.t.6.exotic	survey.1.Satisfaction_Trees_6	3.12	1.01	0.02	
s.t.7.diverse	survey.1.Satisfaction_Trees_7	3.49	0.95	0.02	
s.t.8.habitat	survey.1.Satisfaction_Trees_8	3.71	0.93	0.02	

n=223

Supplementary table 4: Statistical properties of the Satisfaction with the management of urban trees scale, using data from the online panel survey in Toronto, Canada, n=223 (see also *Supplementary figure 4*)

Factors and Items	*Names in graph*	*Mean*	*Standard Deviation (SD)*	*Standard Error (SE)*	*Reliability of all items (standardized Cronbach alpha)*
**as average index**		**3.23**	**0.84**	**0.02**	
s.m.1.timely	survey.1.Satisfaction_Mgmt_1	3.22	1.07	0.02	
s.m.2.remove unhealthy	survey.1.Satisfaction_Mgmt_2	3.22	1.10	0.02	
s.m.3.equitable	survey.1.Satisfaction_Mgmt_3	3.37	1.00	0.02	
s.m.4.responsive to requests	survey.1.Satisfaction_Mgmt_4	3.19	0.98	0.02	0.93
s.m.5.invest	survey.1.Satisfaction_Mgmt_5	3.24	0.96	0.02	
s.m.6.engage	survey.1.Satisfaction_Mgmt_6	2.98	1.01	0.02	
s.m.7.maintenance	survey.1.Satisfaction_Mgmt_7	3.24	1.01	0.02	
s.m.8.habitat	survey.1.Satisfaction_Mgmt_8	3.25	0.98	0.02	

n=223

Supplementary table 5: Statistical properties of the Nature Relatedness (NR6) scale, using data from the online panel survey in Toronto, Canada, n=223 (see also *Supplementary figure 5*)

Factors and Items	*Names in graph*	*Mean*	*Standard Deviation (SD)*	*Standard Error (SE)*	*Reliability of all items (standardized Cronbach alpha)*
**as average index**		**2.88**	**0.94**	**0.02**	
nr6.1	survey.1.NatureRelatedness_1	3.21	1.25	0.03	
nr6.2	survey.1.NatureRelatedness_2	2.78	1.12	0.03	
nr6.3	survey.1.NatureRelatedness_3	3.01	1.21	0.03	0.88
nr6.4	survey.1.NatureRelatedness_4	2.64	1.25	0.03	
nr6.5	survey.1.NatureRelatedness_5	2.83	1.19	0.03	
nr6.6	survey.1.NatureRelatedness_6	2.84	1.15	0.03	

n=223

Supplementary table 6: Greenness profile for the aggregated data from the online panel survey that had postal codes within the City of Toronto, n=223 (see *Methods* in main text)

Measures	*Survey data*				
	Mean [1]	*SD*	*SE*	*Max*	*Min*
VGVI at 500m	0.16	0.08	0.01	0.45	0.01
300m	0.16	0.07	0.00	0.42	0.02
100m	0.16	0.07	0.00	0.40	0.02
NDVI at 500m	0.35	0.10	0.01	0.61	0.02
300m	0.36	0.10	0.01	0.60	0.05
100m	0.36	0.09	0.01	0.61	0.07
Canopy cover at 500m	0.25	0.12	0.01	0.60	0.00
300m	0.26	0.11	0.01	0.56	0.02
100m	0.26	0.11	0.01	0.56	0.04

n=223
1. The mean of a variable is a means of means, given that each measure is calculated as a mean at the individual location level (see *Methods*, main text)

Supplementary table 7: Generalized linear models for listed variables and their association with the level of satisfaction with the management of urban trees controlling for cognitive, social-ecological context, and demographic factors in Toronto, Canada. Statistically significant values in **bold** (n = 223).

Modelled variable	*Estimate*	*95% confidence interval (CI) (lower, higher)*	*Significance (p-value)*	*Null deviance (D)*	*Residual deviance (D)*	*AIC*
VGVI at 500m	1.17	(-0.69, 2.03)	0.22	156.8	143.2	558.5
300m	0.96	(-0.74, 2.65)	0.27	156.8	143.4	558.8
100m	0.42	(-1.21, 2.05)	0.61	156.8	144.1	559.8
NDVI at 500m	0.81	(-0.45, 2.07)	0.21	156.8	143.2	558.4
300m	0.56	(-0.62, 1.74)	0.36	156.8	143.7	559.2
100m	0.44	(-0.72, 1.59)	0.46	156.8	143.9	559.5
Canopy cover at 500m	0.77	(-0.37, 1.91)	0.19	156.8	143.1	558.2
300m	0.63	(-0.44, 1.69)	0.25	156.8	143.4	558.7
100m	0.27	(-0.76, 1.30)	0.61	156.8	144.1	559.8

Each variable is modelled independently at the individual response level. The models control for the following: level of nature relatedness (NR6); level of knowledge of trees; tree in front of home; years in neighbourhood; age (median); Canadian born; English-as-Second-Language; owns a house; education: university degree; ethnicity: white; gender: female; and belongs to an environmental organization (details in Supplements 4-5).

Significance codes: <0.001 ***, <0.01 **, <0.05 *

Supplementary references

References in this supplementary material

1. Schultz, P.W., Shriver, C., Tabanico, J.J., & Khazian, A.M. Implicit connections with nature. *J Environ Psychol* **24,** 31-42 (2004).
2. Schultz, P.W. The structure of environmental concern: Concern for self, other people, and the biosphere. *J Environ Psychol* **21,** 327-339 (2001).
3. Stern, P.C., Kalof, L., Dietz, T., & Guagnano, G.A. Values, beliefs, and proenvironmental action: Attitude formation toward emergent attitude objects. *J Appl Soc Psychol* **25,** 1611-1636 (1995).
4. Schultz, P.W., & Zelezny, L. Values as predictors of environmental attitudes: Evidence for consistency across 14 countries. *J Environ Psychol* **19,** 255-265 (1999).
5. Nordlund, A.M., & Garvill, J. Effects of values, problem awareness, and personal norm on willingness to reduce personal car use. *J Environ Psychol* **23,** 339-347 (2003).
6. Dietz, T., Fitzgerald, A., & Shwom, R. Environmental values. *Ann Rev Environ Resour* **30,** 335-372 (2005).
7. de Groot, R.S., & Steg, M. Value orientations to explain beliefs related to environmental significant behavior: How to measure egoistic, altruistic, and biospheric value orientations. *Environ Behav* **40,** 330-354 (2007).
8. Ford, R.M., Williams, K.J.H., Smith, E.L., & Bishop, I.D. Beauty, belief, and trust: Toward a model of psychological processes in public acceptance of forest management. *Environ Behav* **46,** 476-506 (2012).
9. Heberlein, T.A. *Navigating environmental attitudes* (Oxford University Press, New York, NY, USA, 2012).
10. Whitburn, J., Linklater, W.L., & Milfont, T.L. Exposure to urban nature and tree planting are related to pro-environmental behavior via connection to nature, the use of nature for psychological restoration, and environmental attitudes. *Environ Behav* **51,** 787-810 (2019).
11. Pickett, S.T.A., Cadenasso, M.L., Grove, J.M., Boone, C.G., Groffman, P.M., Irwin, E., Kaushal, S.S., Marshall, V., McGrath, B.P., Nilon, C.H., Pouyat, R.V., Szlavecz, K., Troy, A., & Warren, P. Urban ecological systems: Scientific foundations and a decade of progress. J Environ Manage 92, 331-362 (2011).
