# Supplementary material for: Satisfaction with urban trees associates with tree canopy cover and tree visibility around the home
Source: NPJ Urban Sustain. 2023 Jun 23;3(1):37. doi: 10.1038/s42949-023-00119-8 (PMC11041773; doi:10.1038/s42949-023-00119-8)
Supplement: Supplementary file 2 — Reporting Summary [file 42949_2023_119_MOESM2_ESM.pdf]

Corresponding author(s): Camilo Ordonez Barona
Last updated by author(s): Jun 6, 2023

Reporting Summary

Nature Portfolio wishes to improve the reproducibility of the work that we publish. This form provides structure for consistency and transparency in reporting. For further information on Nature Portfolio policies, see our Editorial Policies and the Editorial Policy Checklist.

Statistics

For all statistical analyses, confirm that the following items are present in the figure legend, table legend, main text, or Methods section.

n/a	Confirmed	
☐	☒	The exact sample size (*n*) for each experimental group/condition, given as a discrete number and unit of measurement
☒	☐	A statement on whether measurements were taken from distinct samples or whether the same sample was measured repeatedly
☐	☒	The statistical test(s) used AND whether they are one- or two-sided<br>*Only common tests should be described solely by name; describe more complex techniques in the Methods section.*
☐	☒	A description of all covariates tested
☐	☒	A description of any assumptions or corrections, such as tests of normality and adjustment for multiple comparisons
☐	☒	A full description of the statistical parameters including central tendency (e.g. means) or other basic estimates (e.g. regression coefficient) AND variation (e.g. standard deviation) or associated estimates of uncertainty (e.g. confidence intervals)
☐	☒	For null hypothesis testing, the test statistic (e.g. *F*, *t*, *r*) with confidence intervals, effect sizes, degrees of freedom and *P* value noted<br>*Give P values as exact values whenever suitable.*
☒	☐	For Bayesian analysis, information on the choice of priors and Markov chain Monte Carlo settings
☒	☐	For hierarchical and complex designs, identification of the appropriate level for tests and full reporting of outcomes
☐	☒	Estimates of effect sizes (e.g. Cohen's *d*, Pearson's *r*), indicating how they were calculated

Our web collection on statistics for biologists contains articles on many of the points above.

Software and code

Policy information about availability of computer code

Data collection	No software was used to collect data.
Data analysis	All uses of software for analyses are openly stated in the main article text.

For manuscripts utilizing custom algorithms or software that are central to the research but not yet described in published literature, software must be made available to editors and reviewers. We strongly encourage code deposition in a community repository (e.g. GitHub). See the Nature Portfolio guidelines for submitting code & software for further information.

Data

Policy information about availability of data

All manuscripts must include a data availability statement. This statement should provide the following information, where applicable:

- Accession codes, unique identifiers, or web links for publicly available datasets
- A description of any restrictions on data availability
- For clinical datasets or third party data, please ensure that the statement adheres to our policy

The remote sensing data, including base files, shape files, and modelling techniques, used and/or analysed during the current study (i.e., greenness measures) are available publicly and online, included in this published article and its supplementary information files, and/or available from the corresponding author on reasonable request. The social datasets generated and/or analysed during the current study (i.e., survey responses) are not publicly available due to restrictions imposed by the institutional ethics review board, as study participants did not consent to sharing this information. These social datasets are available from the

corresponding author on reasonable request without any personal, temporal, or locational information to ensure confidentiality and anonymity of the research participants. Nonetheless, enough details about these datasets are included in this published article in aggregated form in the supplementary material.

Research involving human participants, their data, or biological material

Policy information about studies with human participants or human data. See also policy information about sex, gender (identity/presentation), and sexual orientation and race, ethnicity and racism.

Reporting on sex and gender	Gender, but not sex, was considered in the study design. Findings are not discriminated by gender, but account for gender variables because previous literature had demonstrated the influence of binary gender categories (i.e., female-male) upon perceptions about nature. Gender was self-reported. Gender data were collected through an online panel survey. Gender data are not publicly available due to restrictions imposed by the institutional ethics review board, as study participants did not consent to sharing this information. Informed consent was obtained from participants to collect gender data. Data on the demographic sample profile, including gender, are provided in aggregated form in the supplementary material.
Reporting on race, ethnicity, or other socially relevant groupings	Ethnicity was considered in the study design. Ethnicity data were collected according to the classifications of ethnicity used by Statistics Canada (references in main text of article). Findings are not discriminated by ethnicity, but account for ethnicity variables because previous literature had demonstrated the influence of binary ethnicity categories (i.e., white vs. non-white) upon perceptions about nature. Ethnicity was self-reported. Ethnicity data were collected through an online panel survey. Ethnicity data are not publicly available due to restrictions imposed by the institutional ethics review board, as study participants did not consent to sharing this information. Informed consent was obtained from participants to collect ethnicity data. Data on the demographic sample profile, including ethnicity classifications, are provided in aggregated form in the supplementary material.
Population characteristics	Population characteristics included as covariates in the models of this research include years in neighbourhood; age (median); Canadian born; English-as-Second-Language; owns a house; education: university degree; ethnicity: white; gender: female; and belongs to an environmental organization.
Recruitment	Participants were recruited using an online survey panel managed by Asking Canadians® (www.askingcanadians.com). The company has access to more than 1 million participants, or panellists, in Canada. And online panel survey is an internet-based, self-administered data collection technique that is validated by sociodemographic parameters given that it uses an established panel of respondents. The respondents of the survey were self-selected, meaning self-selection bias was part of the study. Self-selection bias cannot be completely avoided in online panel surveys. However, this bias is reduced by using a nominal fee to compensate participants. This fee is typical in electronic panel surveys (see references in main text) and has the goal of reducing self-selection bias.
Ethics oversight	The protocol of this study was approved by the University of Toronto Ethic Review Board, with Ethic Protocol No. 00040945.

Note that full information on the approval of the study protocol must also be provided in the manuscript.

Field-specific reporting

Please select the one below that is the best fit for your research. If you are not sure, read the appropriate sections before making your selection.

☐ Life sciences ☒ Behavioural & social sciences ☐ Ecological, evolutionary & environmental sciences

For a reference copy of the document with all sections, see nature.com/documents/nr-reporting-summary-flat.pdf

Behavioural & social sciences study design

All studies must disclose on these points even when the disclosure is negative.

Study description	Quantative
Research sample	Panelists in the online panel managed by Asking Canadians® (www.askingcanadians.com), who are also residents of the city of Toronto
Sampling strategy	Systematic, random, and probabilistic sampling
Data collection	Online panel survey
Timing	May 25, 2021 – June 11, 2021
Data exclusions	No data were excluded from the analysis
Non-participation	No participant dropped out or declined participation given the panel nature of the survey
Randomization	not applicable

Reporting for specific materials, systems and methods

We require information from authors about some types of materials, experimental systems and methods used in many studies. Here, indicate whether each material, system or method listed is relevant to your study. If you are not sure if a list item applies to your research, read the appropriate section before selecting a response.

Materials & experimental systems

n/a	Involved in the study
☒	☐ Antibodies
☒	☐ Eukaryotic cell lines
☒	☐ Palaeontology and archaeology
☒	☐ Animals and other organisms
☒	☐ Clinical data
☒	☐ Dual use research of concern
☒	☐ Plants

Methods

n/a	Involved in the study
☒	☐ ChIP-seq
☒	☐ Flow cytometry
☒	☐ MRI-based neuroimaging
